# Adenomyosis and Myomata: Risks, Problems, and Complications in Diagnosis and Therapy of Adenomyosis and Myomata

**DOI:** 10.1155/2018/5952460

**Published:** 2018-08-06

**Authors:** Rudy Leon De Wilde, Markus Wallwiener, Attilio Di Spiezio Sardo, Vasilios Tanos, Sven Becker

**Affiliations:** ^1^Department of Gynecology, Obstetrics and Gynecological Oncology, University Hospital for Gynecology, Pius-Hospital Oldenburg, Oldenburg, Germany; ^2^Department of Obstetrics and Gynecology, University of Heidelberg, Heidelberg, Germany; ^3^Department of Public Health, University of Naples “Federico II”, Naples, Italy; ^4^St. George's Medical School, Nicosia University, Aretaeio Hospital, Nicosia, Cyprus; ^5^Department of Obstetrics and Gynecology, Goethe University Frankfurt, Frankfurt, Germany

The goal of this special issue is to address research concerning risks and problems related to the diagnosis and therapy of adenomyosis and myomata. During the last years, there have been several controversies in the scientific community regarding these topics. The original papers gathered in this special issue highlight and inform the readers about the innovations made in this field.

The complex pathogenesis shows that there are multiple biogenetical and multifactorial aspects influencing the etiology and growth of myomata. Furthermore, the existence of adenomyosis seems to be coupled with molecular differences in myometrial receptors. One of the examples here is the significant increase in GPER expression in case of adenomyosis (J. Li et al.).

Predictive factors that point towards adenomyosis in a clinical set-up make the necessary differential diagnosis between a myoma and an adenomyoma possible. Combining clinical history and symptoms, gynecological examination and MRI (H. Krentel et al.), and sonographical aspects (V. H. Eisenberg et al.) enhance the certainty in the decision-making process. The impact on the reproductive outcome and influence on endometrial receptivity, embryo implantation, and possible “embryotoxicity” as well as anatomical distortion may interfere throughout the duration of pregnancy and affect the obstetrical outcome (N. F. Vlahos et al.).

Hysteroscopy offers the advantage of visualization of the uterine cavity, giving the option of collecting histological samples under visual control. Possibilities of obtaining diagnostic criteria and performing transcervical treatment in selected cases are also discussed (A. Di Spiezio Sardo et al.). The hysteroscopic morcellation of submucous myomata seems feasible, even in cases of a location in the uterine wall of more than half of the tumor (S. G. Vitale et al.).

Myomas affect, with some variability, all ethnic groups and nearly 50% of all women during their lifetime. While some remain asymptomatic, significant and sometimes life-threatening problems can occur. In most cases surgical therapy cannot be avoided: hysteroscopic or laparoscopic therapy is the gold standard (A. El-Balat et al.).

Apart from the routine surgically induced complications, especially in myomata and adenomyosis, there is a risk of injury to the close by organs and a possibility of tissue spilling causing parasitic myoma, endometriosis, or sarcoma spreading (V. Tanos et al.). Possibilities to avoid this tumor spilling by contained in-bag morcellation with description of bag-related application techniques are reported (S. Rimbach et al.).

Nonsurgical alternatives by high-intensity focused ultrasound, eventually combined with oxytocin administration (T. Lozinski et al.), and GnRH-agonists and antagonists have been used in the treatment of symptomatic uterine fibroids with long-lasting effects (M. Safrai et al.). Selective progesterone receptor modulators add perspectives and open new medication-based treatment options (T. Rabe et al.). In all continuously applied medications, not only early drug related complications, but also problems due to the total dosage of the medication should be taken into account.

As this special issue deals with the most frequent diseases treated by gynaecologists, it clearly should be seen as a further step to engage in research and optimizing treatment modalities. The authors, as key opinion leaders in their field, have shared their thoughts and knowledge with only one purpose: to reach a next level in the standard of care dealing with myomata and adenomyosis.

